# A Sensitive Resonance Rayleigh Scattering Method for Na^+^ Based on Graphene Oxide Nanoribbon Catalysis

**DOI:** 10.1155/2018/4017519

**Published:** 2018-12-04

**Authors:** Haidong Wang, Chongning Li, Yanghe Luo, Zhiliang Jiang

**Affiliations:** ^1^School of Food and Bioengineering, Hezhou University, Hezhou 542899, China; ^2^Key Laboratory of Ecology of Rare and Endangered Species and Environmental Protection, (Guangxi Normal University), Ministry of Education, Guangxi Key Laboratory of Environmental Pollution Control Theory and Technology, Guilin 541004, China

## Abstract

The gold nanoparticle reaction of HAuCl_4_-H_2_O_2_ was very slow under 60°C, and the as-prepared graphene oxide nanoribbons (GONRs) exhibited strong catalysis of the reaction to form gold nanoparticles (AuNP) that appeared a resonance Rayleigh scattering (RRS) peak at 550 nm. Upon addition of potassium pyroantimonate (PA) ligand, it was adsorbed on the GONRs surface to inhibit the catalysis to cause the RRS peak decreasing. When the analyte of Na^+^ was added, the coordination reaction between PA and Na^+^ took place to form the stable complexes of [Na_2_(PA)] to release free GONRs catalyst that resulted in the RRS peak increasing linearly. Accordingly, a new and sensitive RRS method for Na^+^ was established, with a linear range of 0.69-25.8 nmol/L and a detection limit of 0.35 nmol/L Na^+^.

## 1. Introduction

Graphene oxide nanoribbons (GONRs), prepared by the oxidative dissociating of multiwalled carbon nanotubes (MWCNTs), show novel physical, chemical, and catalytic properties and good solubility [1–5] and have been utilized in nanoanalysis. Sun et al. [6] synthesized GONRs based on the decompression of MWCNTs by microwave energy, and a core shell MWCNT/GONR modified glass carbon electrode was fabricated to detect 0.1-8.5 *μ*mol/L ascorbic acid, 0.15-12.15 *μ*mol/L dopamine, and 0.15-11.4 *μ*mol/L uric acid simultaneously, with detection limits of 0.06 *μ*mol/L, 0.08*μ*mol/L and 0.07*μ*mol/L respectively. Zhu et al. [7] prepared a core–shell heterostructure MWCNTs@GONRs by partially unzipping of MWCNTs from longitudinal side with a simple wet chemical strategy and applied it for electrochemical determination of three kinds of polycyclic aromatic amine (PAAs). Zhang et al. [8] carried out PtPd-rGONRs nanocubes by fixing GONRs on the PtPd concave through a hydrothermal process. The PtPd-rGONRs-based electrochemical detection platform can be applied to 0.01-3*μ*g/mL trinitrotoluene (TNT) detection in tap water and lake water samples, with a detection limit of 0.8 ng/mL. Up to date, there is rare report about using GONRs to detect sodium by resonance Rayleigh scattering (RRS) method.

RRS is simple, rapid, and sensitive and has been used in a wide range of applications in different fields such as biochemistry, analytical chemistry, and nanomaterial research [9–14]. Liang et al. [15] reported a RRS-ET method for the analysis of fluorine. Fluorine ions react with fluorine reagent (FR) and La(III) to generate blue ternary complex that exhibited strong absorption at about 370 nm. Upon addition of graphene oxide/nanogold (GO/NG) as RRS spectral probe with strong RRS peak at 370 nm, and the RRS intensity decreased with the increase of fluorine ion concentration due to the RRS energy transfer (RRS-ET). Its linear range was 6.0×10^−8^-1.3×10^−5^mol/L, with a detection limit of 3.0 × 10^−8^mol/L. Wang et al. [16] prepared graphene oxide/gold nanoparticle (GO/GN) composites by citrate reduction that exhibited a strong resonance RS (RRS) peak at 370 nm, and 0.025-5 mM KIO_3_ and 0.5-100 mM H_2_O_2_ can be determined, respectively. Yang et al. [17] reported a RRS method for simultaneous determination of D- L-tryptophan chiral enantiomer complexed with *β*-Cyclodextrin. Sodium ions play an important role in life activities. Most of it exists in the extracellular and bone; it plays a role in regulating the body's osmotic pressure, acid-base balance, muscle, and heart activity. The imbalance of sodium ions in the body can cause some discomfort, including convulsions, hypotension, vomiting, heart failure, and kidney failure. Therefore, the detection of Na^+^ has become an important issue. The common methods for the determination of Na^+^ are potentiometric titration [18], flame atomic emission spectrometry [19], temperature titration [20], atomic absorption spectrometry [21], ion chromatography [22], and inductively coupled plasma mass spectrometry [23]. Some of these methods have the advantages of low sensitivity, complicated operation, large ion interference, and many affecting factors. In this paper, the as-prepared GONRs were used as catalysis, and a rapid and sensitive RRS method was established for detecting Na^+^, based on the ligand regulating GONRs catalytic activity.

## 2. Results and Discussion

### 2.1. Analytical Principle

**Figure 1:**
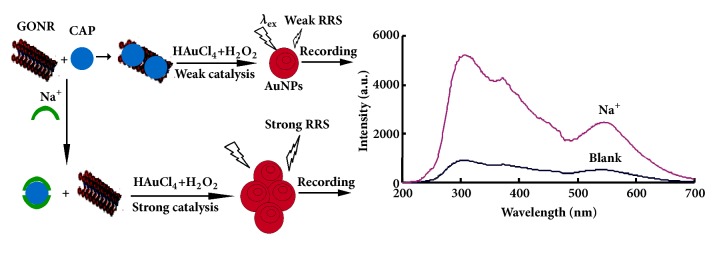
Principle of the RRS detection of Na^+^.

Under the selected conditions, GONRs had strong catalysis on the reduction of HAuCl_4_ by H_2_O_2_ which was very slow without a catalyst. With the increase of GONRs concentration, the reaction was obviously enhanced. The concentration of formed gold nanoparticles in the system was increased, which showed a strong RRS peak. Potassium pyroantimonate (PA) binding on GONRs surface had an inhibitory effect on the catalysis of GONRs. Moreover, Na^+^ could react with CAP to form stable complexes Na_2_CAP, thus releasung the binding of CAP to GONRs and restoring the catalysis of GONRs to cause the RRS peak increasing. Accordingly, a new RRS method for Na^+^ by ligand regulating GONRs catalytic activity was established (Figure 1).

### 2.2. RRS Spectra

**Figure 2:**
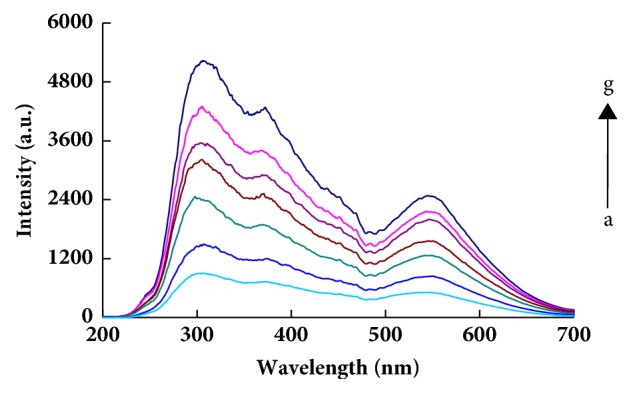
RRS spectra of CH_3_CH_2_OH-Na^+^-PA-GONR-H_2_O_2_-HAuCl_4_ system. a:1.13 mol/L CH_3_CH_2_OH+ 3.14 *μ*mol/L CAP+0.193 mmol/L HAuCl_4_ +3.3 mmol/L H_2_O_2_+23.8 ng/mL GONR; b:a+0.69 nmol/L Na^**+**^; c:a+4.3 nmol/L Na^**+**^; d:a+8.6 nmol/L Na^**+**^; e:a+12.9 nmol/L Na^**+**^; f:a+17.2 nmol/L Na^**+**^; g:a+25.8 nmol/L Na^**+**^.

The CH_3_CH_2_OH-Na^+^-PA-GONR-H_2_O_2_-HAuCl_4_ system exhibited three RRS peaks at 305nm, 370 nm, and 550 nm that are ascribing to surface plasmon resonance effect of AuNPs (Figure 2). With the concentration of Na^+^ increased, the RRS peaks increased linearly. Although both peaks at 305nm and 370 nm are more sensitive than the peak at 550 nm, the latter is free from interference of benzene compounds and was selected for determination of sodium ions. While the CH_3_CH_2_OH-Na^+^-PA system has two weak peaks at 295 nm and 370 nm, and the RRS signals increased with Na^+^ concentration increase slowly (Fig.  S1). Therefore, the contribution of [Na_2_(PA)] on the system can be excluded. The CH_3_CH_2_OH-Na^+^-PA-GO-H_2_O_2_-HAuCl_4_-VB4r system generated the characteristic nanogold peaks at 370 nm and 545 nm (Fig.  S2). The RRS spectra of different analysis systems were studied, and the intensity of RRS spectra increased gradually with the increase of the amount of analyte. The binding of GONR and PA inhibit the catalysis when there was no Na^+^ (Fig.  S3, S4). In a certain range, with the increase of GO, GONR concentration, the reaction of H_2_O_2_-HAuCl_4_ system was enhanced, so the concentration of nanoparticles generated by the reaction increased and the RRS signals of the system gradually increased (Fig.  S5, S6).

### 2.3. Absorption Spectra

**Figure 3:**
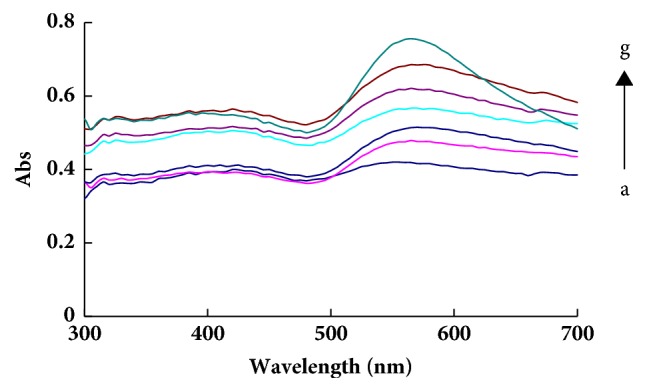
Absorption spectra of CH_3_CH_2_OH-Na^+^-PA-GONR-H_2_O_2_-HAuCl_4_ system. a:1.13 mol/L CH_3_CH_2_OH+ 3.14 *μ*mol/L CAP+0.193 mmol/L HAuCl_4_ +3.3 mmol/L H_2_O_2_+23.8 ng/mL GONR; b:a+0.69 nmol/L Na^**+**^; c:a+4.3 nmol/L Na^**+**^; d:a+8.6 nmol/L Na^**+**^; e:a+12.9 nmol/L Na^**+**^; f:a+17.2 nmol/L Na^**+**^; g:a+25.8 nmol/L Na^**+**^.

According to the procedure, the UV-vis absorption spectra of analytical systems were recorded. There was an absorption peak at 580 nm of the CH_3_CH_2_OH-Na^+^-PA–GONR/GO-H_2_O_2_-HAuCl_4_ system, and the peak gradually increased and narrowed with the increase of Na^+^ concentration in a certain range (Figure 3, Fig. S7).The binding of GONR and PA inhibits the catalysis when there was no Na^+^ (Fig.  S8, Fig. S9). As the concentration of GONR/GO increased, the catalytic activity increased and more gold nanoparticles were formed, and the absorption of the system gradually increased (Fig.  S10, Fig.  S11).

### 2.4. Catalysis and Inhibition

According to the experimental conditions, GO/GONR can catalyze the reaction of H_2_O_2_-HAuCl_4_ to form gold nanoparticles. In a certain range of concentration, the catalytic ability was enhanced with the catalyst increase and the formed gold nanoparticles were increased. The RRS intensity of 550 nm increases linearly and the corresponding UV absorbance has a certain degree of enhancement to catalyst concentration. At the same time, the catalytic effects of different quantum dots were compared. (Table 1), GONR-H_2_O_2_-HAuCl_4_ has the strongest catalytic ability. The PA attaches to the surface of the catalyst, preventing contact of the catalyst with the reactants when PA is added to the system. As the amount of PA increased, the catalytic RRS/UV effect was attenuated, in which the system ΔI_550nm_ was negatively correlated with PA concentration (Table 1).

### 2.5. Scanning Electron Microscopy (SEM)

**Figure 4:**
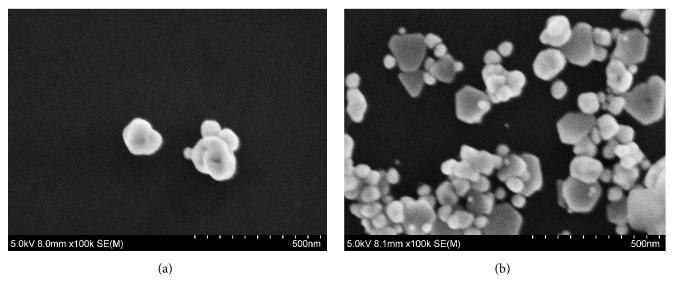
**SEM of the analysis system. **a:1.13 mol/L CH_3_CH_2_OH+ 3.14 *μ*mol/L PA+0.193 mmol/L HAuCl_4_ +3.3 mmol/L H_2_O_2_+23.8 ng/mL GONR; b:a+17.2 nmol/L Na^**+**^.

According to the selected conditions, 0.1mL reaction solution was taken and diluted 100 times in a 10 mL centrifuge tube. Then 10 *μ*L sample solution was dropped onto the silicon wafer and naturally dried, and SEM of the sample was obtained. For the Na^+^-PA-GONR-H_2_O_2_-HAuCl_4_ system, the binding of GONR and PA inhibits the catalysis when there was no Na^+^. So the reaction of H_2_O_2_ reducing HAuCl_4_ at 60°C was slower, and the produced nanogold in the solution was less (Figure 4(a)), with an average diameter of 150 nm. When Na^+^ was added, GONR was not completely bound and had some catalysis, resulting in a certain amount of nanogold with irregularly shaped particles with the average diameter of about 100 nm (Figure 4(b)).

### 2.6. Optimization of Analytical Conditions

The analytical conditions were optimized, respectively (Fig.  S12). The effect of GONRs concentration on the system was investigated and the ΔI values increased rapidly at first and reached the maximum when the concentration of GONR was 15.8 *μ*g/mL. So, 15.8 *μ*g/mL GONR was chosen. The ΔI values of SERS increased rapidly at first and decreased after the maximum with CAP concentration increased. According to the result, 3.14 *μ*mol/L CAP was chosen. H_2_O_2_ was a reducer for the redox reaction and when the concentration of H_2_O_2_ was 3.3 mmol/L, ΔI was the largest. So 3.3 mmol/L H_2_O_2_ was chosen. The effect of HAuCl_4_ concentration was investigated according to the procedure and when HAuCl_4_ concentration was 0.193 mmol/L, ΔI was the largest. So 0.193 mmol/L HAuCl_4_ was selected. The effect of reaction temperature was investigated in the range of 20-80°C water bath. ΔI enhanced sharply with the temperature increasing at 40-60°C and tended to be stable before and after that. So, a water bath of 60°C was used. The effect of reaction time was investigated. ΔI got the maximum and tended to be stable when the reaction time was 20 min. So, 20 min was chosen.

### 2.7. Working Curve

The working curves between Na^+^ concentration and their corresponding ∆I were plotted for the GONR and GO two analytical systems. Results show that the GONR system was more sensitive than the GO, with a linear range of 0.69-25.8 *μ*mol/L, regression equation of ΔI = 85.4C_Na_+244.1, a coefficient of 0.9712, and detection limit of 0.35 nmol/L Na^+^ and was selected to determine Na^+^ in sample.

### 2.8. Influence of Interfering Ions

The interference of common coexistence on the determination of 17.2 nmol/L Na^+^ was investigated within a relative error of ±10%. Results showed that 100 times Ca^2+^, Ni^2+^, Bi^3+^, NH_4_^+^, SO_4_^2−^, Pb^2+^, Zn^2+^, NO_2_^−^, glutamic acid, aspartic acid, valine, phenylalanine, 80 times Cr^6+^, Mg^2+^, Al^3+^, K^+^, 50 times Ba^2+^, Fe^3+^, Mn^2+^, and 10 times Cu^2+^ had no interference on the determination of Na^+^. Therefore, the method had good selectivity.

### 2.9. Sample Analysis

The drinking water samples were purchased from supermarket and filtered by filter paper. According to the procedure, Na^+^ content was detected in the samples. The RRS results were in agreement with that of atomic absorption spectrometry (AAS), the recovery was 96.5% to 98.2%, and relative standard deviation is 3.3% to 6.7% (Table 2).

## 3. Materials and Methods

### 3.1. Apparatus

A model of Hitachi F-7000 fluorescence spectrophotometer (Hitachi High-Technologies Corporation, Japan) by synchronous scanning technique with a volt of 400 v, excited slit and emission slit of 5 nm, emission filter of 1% T attenuator and *λ*_ex_-*λ*_em_=Δ*λ*=0, a model of TU-1901 double beam UV-visible spectrophotometer (Beijing Purkinje General Instrument Co., Ltd. China), a model of 3K-15 high- speed refrigerated centrifuge (Sigma Co., Germany), a model of 79-1 magnetic stirrer with heating (Zhongda Instrumental Plant, Jiangsu, China), a model of HH-S2 electric hot water bath (Earth Automation Instrument Plant, Jintan, China), a model of Zetasizer Nano nanometer particle size and zeta potential analyzer (Malvern, UK), S-4800 field emission scanning electron microscope (Hitachi High-Technologies Corporation, Japan/Oxford company, UK), and a model of SYZ-550 quartz subboiling distilled water (Crystal Glass Instrument Plant, Jiangsu, China) were used.

### 3.2. Reagents

A 11.8 mmol/L potassium pyroantimonate (PA) solution: 1.5 g of potassium pyroantimonate (AR. Shanghai Reagent Four Hewei Chemical Co., Ltd.) was added to a conical flask before 150 mL distilled water and 0.15 g KOH was added and boiled to dissolve. After cooling to room temperature, the solution was transferred into a 250 mL volumetric flask and added water to the mark. It was diluted 100 times for use. A 2.9 mmol/L HAuCl_4_ (National Pharmaceutical Group Chemical Reagents Company, China), 10 *μ*mol/L VB4r, 10 mol/L H_2_O_2_, KMnO_4_(s), multiwall carbon nanotube (MWCNTs, short, 8-15 nm, 95% purity, length of 0.5-2 *μ*m, 8-15 nm in diameter, No. XFM10, XFNANO, Nanjing), 50% CH_3_CH_2_OH, 50 mmol/L KOH, 129 mmol/L NaCl standard solution, 39.33 *μ*mol/L sodium pyroantimonate solution (118 *μ*mol/L potassium pyroantimonate solution and 129 mmol/L NaCl solution were mixed in a volume ratio of 1:2 according to the desired volume) were prepared. A 1.0 mg grapheme oxide (GO) was dissolved in 100 mL water with ultrasonic treatment to obtain 10 *μ*g/mL GO solution. It was treating for 15 min by ultrasonic process before use. All reagents were of analytical grade and the water was doubly distilled.

GONRs were prepared by the dissociating of MWCNTs. 50 mg MWCNT powder was added to a 50 mL round bottom flask containing 10 mL of concentrated H_2_SO_4_ before reacting for 1 h. Then 300 mg of KMnO_4_ was added to the solution, mixed well, and heated at 60°C for 2 h. Next, the product was poured into 200 mL of ice water containing 5 mL of 30% H_2_O_2_. Then the solution was ultrasonicated for 10 min before centrifuging at 7000 rpm for 10 min. The supernatant was taken and the manganese dioxide generated in the reaction was removed. The final concentration of GONRs solution was 238 *μ*g/mL. It was neutralized with 50 mmol/L NaOH and then diluted to the desired concentration before use.  .

### 3.3. Procedure

A 100 *μ*L 50% CH_3_CH_2_OH, 50 *μ*L 118 *μ*mol/L PA and a certain amount of NaCl standard solutions were added to a 5 mL marked test tube, mixed well, and reacted for 10 min at room temperature. Then a 100 *μ*L 238 ng/mL GONRs, 100 *μ*L 2.9 mmol/L HAuCl_4_, and 50 *μ*L 0.1 mmoL/L H_2_O_2_ solutions were added before diluting to 1.5 mL with water. The mixed solution was placed in a 60°C water bath for reacting 20 min and the reaction was terminated by tap water. The mixture was transferred into a quartz cell, and its RRS spectra were recorded. The RRS intensity I at 550 nm and the blank I_0_ without Na^+^ were recorded, and ΔI = I–I_0_ was calculated.

## 4. Conclusions

Based on the selective reaction of Na^+^ with CAP to control the GONR catalysis of the reduction of HAuCl_4_ with H_2_O_2_ to produce gold nanoparticles with strong RRS effect, a new RRS method for the determination of Na^+^ was established. It has the advantages of simple operation, high sensitivity, and good selectivity.

## Figures and Tables

**Table 1:** Comparing of the catalytic and inhibition characteristics by RRS method.

System	Regress equation	Linear range	Coefficient
GONR	1.58-55.8 ng/mL	Δ*I*_550*nm*_=50.8 C_GONR_ +26. 7	0.9868
GO	6.6-50 ng/mL	Δ*I*_545*nm*_=34.2C_GO_+130.2	0.9768
PA-GONR	0.79-5.5 *μ*mol/L	Δ*I*_550*nm*_=241.8 C_PA_-8.6	0.983
PA-GO	0.79-6.29 *μ*mol/L	Δ*I*_545*nm*_=150.4 C_PA_+130	0.9537

**Table 2:** Analytical results of Na^+^ in samples.

Sample	Detection (ng/L)	Average (ng/L)	Added (ng/L)	Found (ng/L)	Recovery (%)	RSD (%)	Content (mg/L)	AAS (mg/L)
Sample1	205.9, 198.6, 185.9, 211.3, 222.6	204.9	367.6	558.6	97.6	6.7	2.1	2.0
Sample2	156.9, 157.3, 144.2, 152.1, 148.6	151.8	367.6	510.2	98.2	3.6	1.5	1.6
Sample3	101.3, 99.7, 102.5, 106.9, 98.1	101.7	367.6	453.1	96.5	3.3	1.8	1.9

## Data Availability

The data [see [1–23]] used to support the findings of this study are included within the article.

## Supplementary Materials

Supplementary Materials
** Fig.S1 RRS spectra of** **C** **H**_3_ **C** **H**_2_ **O** **H**-**N****a**^+^-**P****A**** system.** a: 1.13 mol/L CH_3_CH_2_OH + 3.14 *μ*mol/L CAP; b:a+0.69 nmol/L Na^**+**^; c:a+4.3 nmol/L Na^**+**^; d:a+8.6 nmol/L Na^**+**^; e:a+12.9 nmol/L Na^**+**^; f:a+17.2 nmol/L Na^**+**^; g:a+25.8 nmol/L Na^**+**^.** Fig.S2 RRS spectra of ****C****H**_3_**C****H**_2_**O****H**-**N****a**^+^-**P****A**-**G****O**-**H**_2_**O**_2_-**H****A****u****C****l**_4_** system.** a:1.13 mol/L CH_3_CH_2_OH+ 3.14 *μ*mol/L CAP+0.193 mmol/L HAuCl_4_ +3.3 mmol/L H_2_O_2_+50 ng/mL GO; b:a+0.86 nmol/L Na^**+**^; c:a+4.3 nmol/L Na^**+**^; d:a+8.6 nmol/L Na^**+**^; e:a+12.9 nmol/L Na^**+**^; f:a+17.2 nmol/L Na^**+**^; g:a+25.8 nmol/L Na^**+**^.** Fig.S3 RRS of ****C****H**_3_**C****H**_2_**O****H**-**G****O****N****R**-**H**_2_**O**_2_-**H****A****u****C****l**_4_** system.** a:1.13 mol/L CH_3_CH_2_OH+ 3.14 *μ*mol/L CAP+0.193 mmol/L HAuCl_4_ +3.3 mmol/L H_2_O_2_+50ng/mL GO; b:a+0.79 *μ*mol/L PA; c:a+2.36 *μ*mol/L PA; d:a+3.14 *μ*mol/L PA; e:a+4.0 *μ*mol/L PA; f:a+4.72 *μ*mol/L PA; g:a+5.5 *μ*mol/L PA.** Fig.S4 RRS of ****C****H**_3_**C****H**_2_**O****H**  -**G****O**-**H**_2_**O**_2_-**H****A****u****C****l**_4_** system.** a:1.13 mol/L CH_3_CH_2_OH+ 3.14 *μ*mol/L CAP+0.193 mmol/L HAuCl_4_ +3.3 mmol/L H_2_O_2_+50ng/mL GO; b:a+0.79 *μ*mol/L PA; c:a+2.36 *μ*mol/L PA; d:a+3.14 *μ*mol/L PA; e:a+4.72 *μ*mol/L PA; f:a+5.5 *μ*mol/L PA; g:a+6.29 *μ*mol/L PA.** Fig.S5 RRS spectra of ****G****O****N****R**-**H**_2_**O**_2_-**H****A****u****C****l**_4_** system.** a:1.13 mol/L CH_3_CH_2_OH +0.193 mmol/L HAuCl_4_ +3.3 mmol/L H_2_O_2_; b:a+ 1.58 ng/mL GONR; c:a+3.17 ng/mL GONR; d:a+7.93 ng/mL GONR; e:a+11.1 ng/mL GONR; f:a+14.3 ng/mL GONR; g:a+15.8 ng/mL GONR.** Fig.S6 RRS spectra of GO-****H**_2_**O**_2_-**H****A****u****C****l**_4_** system.** a:1.13 mol/L CH_3_CH_2_OH+0.193 mmol/L HAuCl_4_ +3.3 mmol/L H_2_O_2_; b:a+ 6.6 ng/mL GO; c:a+13.3 ng/mL GO; d:a+16.6 ng/mL GO; e:a+20 ng/mL GO; f:a+26.6 ng/mL GO; g:a+50 ng/mL GO.** Fig. S7 UV of ****C****H**_3_**C****H**_2_**O****H**-**N****a**^+^-  **P****A**  -**G****O**-**H**_2_**O**_2_-**H****A****u****C****l**_4_** system.** a:1.13 mol/L CH_3_CH_2_OH+ 3.14 *μ*mol/L CAP+0.193 mmol/L HAuCl_4_ +3.3 mmol/L H_2_O_2_+50ng/mL GO; b:a+0.86 nmol/L Na^**+**^; c:a+4.3 nmol/L Na^**+**^; d:a+12.9 nmol/L Na^**+**^; e:a+17.2 nmol/L Na^**+**^; f:a+25.8 nmol/L Na^**+**^.** Fig. S8 UV of ****C****H**_3_**C****H**_2_**O****H**-**G****O****N****R**-**H**_2_**O**_2_-**H****A****u****C****l**_4_** system.** a:1.13 mol/L CH_3_CH_2_OH+ 3.14 *μ*mol/L CAP+0.193 mmol/L HAuCl_4_ +3.3 mmol/L H_2_O_2_+50ng/mL GO; b:a+0.79 *μ*mol/L PA; c:a+2.36 *μ*mol/L PA; d:a+3.14 *μ*mol/L PA; e:a+4.0 *μ*mol/L PA; f:a+4.72 *μ*mol/L PA; g:a+5.5 *μ*mol/L PA.** Fig. S9 UV of ****C****H**_3_**C****H**_2_**O****H**  -**G****O**-**H**_2_**O**_2_-**H****A****u****C****l**_4_** system.** a:1.13 mol/L CH_3_CH_2_OH+ 3.14 *μ*mol/L CAP+0.193 mmol/L HAuCl_4_ +3.3 mmol/L H_2_O_2_+50ng/mL GO; b:a+0.79 *μ*mol/L PA; c:a+2.36 *μ*mol/L PA; d:a+3.14 *μ*mol/L PA; e:a+4.72 *μ*mol/L PA; f:a+5.5 *μ*mol/L PA; g:a+6.29 *μ*mol/L PA.** Fig. S10 UV of ****C****H**_3_**C****H**_2_**O****H**-**G****O****N****R**-**H**_2_**O**_2_-**H****A****u****C****l**_4_** system.** a:1.13 mol/L CH_3_CH_2_OH +0.193 mmol/L HAuCl_4_ +3.3 mmol/L H_2_O_2_; b:a+ 1.58 ng/mL GONR; c:a+6.34 ng/mL GONR; d:a+9.52 ng/mL GONR; e:a+12.6 ng/mL GONR; f:a+15.8 ng/mL GONR; g:a+23.8 ng/mL GONR.** Fig.S11 UV spectra of ****C****H**_3_**C****H**_2_**O****H**-**G****O**-**H**_2_**O**_2_-**H****A****u****C****l**_4_** system.** a:1.13 mol/L CH_3_CH_2_OH+0.193 mmol/L HAuCl_4_ +3.3 mmol/L H_2_O_2_; b:a+ 6.6 ng/mL GO; c:a+13.3 ng/mL GO; d:a+20 ng/mL GO; e:a+26.6 ng/mL GO; f:a+33.3 ng/mL GO.** Fig.S12a The effect of GONR concentration.** 1.13 mol/L CH_3_CH_2_OH+17.2 nmol/L Na^+^ + 3.14 *μ*mol/L PA + 0.193 mmol/L HAuCl_4_ + 3.3 mmol/L H_2_O_2_.** Fig. S12b The effect of PA concentration. **1.13 mol/L CH_3_CH_2_OH+17.2 nmol/L Na^**+**^+ 0.193 mmol/L HAuCl_4_ +3.3 mmol/L H_2_O_2_+23.8 ng/mL GONR.** Fig. S12c The effect of ****H**_2_**O**_2_** concentration.** 1.13 mol/L CH_3_CH_2_OH+17.2 nmol/L Na^**+**^+ 3.14 *μ*mol/L PA+0.193 mmol/L HAuCl_4_ + 23.8 ng/mL GONR.** Fig. S12d The effect of ****H****A****u****C****l**_4_** concentration.** 1.13 mol/L CH_3_CH_2_OH+17.2 nmol/L Na^**+**^+ 3.14 *μ*mol/L PA+ 3.3 mmol/L H_2_O_2_+23.8 ng/mL GONR.** Fig. S12e The effect of temperature.** 1.13 mol/L CH_3_CH_2_OH+17.2 nmol/L Na^**+**^+ 3.14 *μ*mol/L PA+0.193 mmol/L HAuCl_4_ +3.3 mmol/L H_2_O_2_+23.8 ng/mL GONR.** Fig. S12f The effect of reaction time.** 1.13 mol/L CH_3_CH_2_OH+17.2 nmol/L Na^**+**^+ 3.14 *μ*mol/L PA+0.193 mmol/L HAuCl_4_ +3.3 mmol/L H_2_O_2_+23.8 ng/mL GONR.
