# Supplementary material for: A Sensitive Resonance Rayleigh Scattering Method for Na+ Based on Graphene Oxide Nanoribbon Catalysis
Source: Int J Anal Chem. 2018 Dec 4;2018:4017519. doi: 10.1155/2018/4017519 (PMC6304511; doi:10.1155/2018/4017519)
Supplement: Supplementary Materials — Fig.S1 RRS spectra of C H 3 C H 2 O H-Na+-PA system. a: 1.13 mol/L CH3CH2OH + 3.14 μmol/L CAP; b:a+0.69 nmol/L Na+; c:a+4.3 nmol/L Na+; d:a+8.6 nmol/L Na+; e:a+12.9 nmol/L Na+; f:a+17.2 nmol/L Na+; g:a+25.8 nmol/L Na+. Fig.S2 RRS spectra of CH3CH2OH-Na+-PA-GO-H2O2-HAuCl4 system. a:1.13 mol/L CH3CH2OH+ 3.14 μmol/L CAP+0.193 mmol/L HAuCl4 +3.3 mmol/L H2O2+50 ng/mL GO; b:a+0.86 nmol/L Na+; c:a+4.3 nmol/L Na+; d:a+8.6 nmol/L Na+; e:a+12.9 nmol/L Na+; f:a+17.2 nmol/L Na+; g:a+25.8 nmol/L Na+. Fig.S3 RRS of CH3CH2OH-GONR-H2O2-HAuCl4 system. a:1.13 mol/L CH3CH2OH+ 3.14 μmol/L CAP+0.193 mmol/L HAuCl4 +3.3 mmol/L H2O2+50ng/mL GO; b:a+0.79 μmol/L PA; c:a+2.36 μmol/L PA; d:a+3.14 μmol/L PA; e:a+4.0 μmol/L PA; f:a+4.72 μmol/L PA; g:a+5.5 μmol/L PA. Fig.S4 RRS of CH3CH2OH -GO-H2O2-HAuCl4 system. a:1.13 mol/L CH3CH2OH+ 3.14 μmol/L CAP+0.193 mmol/L HAuCl4 +3.3 mmol/L H2O2+50ng/mL GO; b:a+0.79 μmol/L PA; c:a+2.36 μmol/L PA; d:a+3.14 μmol/L PA; e:a+4.72 μmol/L PA; f:a+5.5 μmol/L PA; g:a+6.29 μmol/L PA. Fig.S5 RRS spectra of GONR-H2O2-HAuCl4 system. a:1.13 mol/L CH3CH2OH +0.193 mmol/L HAuCl4 +3.3 mmol/L H2O2; b:a+ 1.58 ng/mL GONR; c:a+3.17 ng/mL GONR; d:a+7.93 ng/mL GONR; e:a+11.1 ng/mL GONR; f:a+14.3 ng/mL GONR; g:a+15.8 ng/mL GONR. Fig.S6 RRS spectra of GO-H2O2-HAuCl4 system. a:1.13 mol/L CH3CH2OH+0.193 mmol/L HAuCl4 +3.3 mmol/L H2O2; b:a+ 6.6 ng/mL GO; c:a+13.3 ng/mL GO; d:a+16.6 ng/mL GO; e:a+20 ng/mL GO; f:a+26.6 ng/mL GO; g:a+50 ng/mL GO. Fig. S7 UV of CH3CH2OH-Na+- PA -GO-H2O2-HAuCl4 system. a:1.13 mol/L CH3CH2OH+ 3.14 μmol/L CAP+0.193 mmol/L HAuCl4 +3.3 mmol/L H2O2+50ng/mL GO; b:a+0.86 nmol/L Na+; c:a+4.3 nmol/L Na+; d:a+12.9 nmol/L Na+; e:a+17.2 nmol/L Na+; f:a+25.8 nmol/L Na+. Fig. S8 UV of CH3CH2OH-GONR-H2O2-HAuCl4 system. a:1.13 mol/L CH3CH2OH+ 3.14 μmol/L CAP+0.193 mmol/L HAuCl4 +3.3 mmol/L H2O2+50ng/mL GO; b:a+0.79 μmol/L PA; c:a+2.36 μmol/L PA; d:a+3.14 μmol/L PA; e:a+4.0 μmol/L PA; f:a+4.72 μmol/L PA; g:a+5.5 μmol/L PA. Fig. S9 UV of CH3CH2OH -GO-H2O2-HAuCl4 system. a [file 4017519.f1.doc]

**A sensitive resonance Rayleigh scattering method for Na^+^ based on graphene oxide nanoribbon catalysis**

Haidong Wang^1,2^, Chongning Li^1,2^, Yanghe Luo^1*^ and Zhiliang Jiang^*1,2^

^1^ School of Food and Bioengineering, Hezhou University, Hezhou 542899, China;

^2^ Key Laboratory of Ecology of Rare and Endangered Species and Environmental Protection, (Guangxi Normal University), Ministry of Education, Guangxi Key Laboratory of Environmental Pollution Control Theory and Technology, Guilin 541004, China; 18074841309@163.com (H.W.); lcn7882342@163.com (C.L.)

***** Correspondence: kira0217@foxmail.com (Y.L.); zljiang@mailbox.gxnu.edu.cn (Z.J.);
Tel.:+86-0773-5846141 (Z.J.)

g

a

**Fig.S1 RRS spectra of CH_3_CH_2_OH-Na^+^-PA system**

a: 1.13 mol/L CH_3_CH_2_OH + 3.14 μmol/L CAP; b:a+0.69 nmol/L Na**^+^**; c:a+4.3 nmol/L Na**^+^**; d:a+8.6 nmol/L Na**^+^**; e:a+12.9 nmol/L Na**^+^**; f:a+17.2 nmol/L Na**^+^**; g:a+25.8 nmol/L Na**^+^.**

g

a

**Fig.S2 RRS spectra of CH_3_CH_2_OH-Na^+^-PA-GO-H_2_O_2_-HAuCl_4_ system**

a:1.13 mol/L CH_3_CH_2_OH+ 3.14 μmol/L CAP+0.193 mmol/L HAuCl_4_ +3.3 mmol/L H_2_O_2_+50 ng/mL GO; b:a+0.86 nmol/L Na**^+^**; c:a+4.3 nmol/L Na**^+^**; d:a+8.6 nmol/L Na**^+^**; e:a+12.9 nmol/L Na**^+^**; f:a+17.2 nmol/L Na**^+^**; g:a+25.8 nmol/L Na**^+^.**

**Fig.S3 RRS of CH_3_CH_2_OH-GONR-H_2_O_2_-HAuCl_4_ system**

a

g

a:1.13 mol/L CH_3_CH_2_OH+ 3.14 μmol/L CAP+0.193 mmol/L HAuCl_4_ +3.3 mmol/L H_2_O_2_+50ng/mL GO; b:a+0.79 μmol/L PA; c:a+2.36 μmol/L PA; d:a+3.14 μmol/L PA; e:a+4.0 μmol/L PA; f:a+4.72 μmol/L PA; g:a+5.5 μmol/L PA**.**

**Fig.S4 RRS of CH_3_CH_2_OH -GO-H_2_O_2_-HAuCl_4_ system**

a

g

a:1.13 mol/L CH_3_CH_2_OH+ 3.14 μmol/L CAP+0.193 mmol/L HAuCl_4_ +3.3 mmol/L H_2_O_2_+50ng/mL GO; b:a+0.79 μmol/L PA; c:a+2.36 μmol/L PA; d:a+3.14 μmol/L PA; e:a+4.72 μmol/L PA; f:a+5.5 μmol/L PA; g:a+6.29 μmol/L PA**.**

g

a

**Fig.S5 RRS spectra of GONR-H_2_O_2_-HAuCl_4_ system**

a:1.13 mol/L CH_3_CH_2_OH +0.193 mmol/L HAuCl_4_ +3.3 mmol/L H_2_O_2_; b:a+ 1.58 ng/mL GONR; c:a+3.17 ng/mL GONR; d:a+7.93 ng/mL GONR ; e:a+11.1 ng/mL GONR; f:a+14.3 ng/mL GONR; g:a+15.8 ng/mL GONR

g

a

**Fig.S6 RRS spectra of GO-H_2_O_2_-HAuCl_4_** **system**

a:1.13 mol/L CH_3_CH_2_OH+0.193 mmol/L HAuCl_4_ +3.3 mmol/L H_2_O_2_ ; b:a+ 6.6 ng/mL GO; c:a+13.3 ng/mL GO; d:a+16.6 ng/mL GO ; e:a+20 ng/mL GO; f:a+26.6 ng/mL GO; g:a+50 ng/mL GO.

**Fig. S7 UV of CH_3_CH_2_OH-Na^+^- PA -GO-H_2_O_2_-HAuCl_4_ system**

f

a

a:1.13 mol/L CH_3_CH_2_OH+ 3.14 μmol/L CAP+0.193 mmol/L HAuCl_4_ +3.3 mmol/L H_2_O_2_+50ng/mL GO; b:a+0.86 nmol/L Na**^+^**; c:a+4.3 nmol/L Na**^+^**; d:a+12.9 nmol/L Na**^+^**; e:a+17.2 nmol/L Na**^+^**; f:a+25.8 nmol/L Na**^+^.**

**Fig. S8 UV of CH_3_CH_2_OH-GONR-H_2_O_2_-HAuCl_4_ system**

a

g

a:1.13 mol/L CH_3_CH_2_OH+ 3.14 μmol/L CAP+0.193 mmol/L HAuCl_4_ +3.3 mmol/L H_2_O_2_+50ng/mL GO; b:a+0.79 μmol/L PA; c:a+2.36 μmol/L PA; d:a+3.14 μmol/L PA; e:a+4.0 μmol/L PA; f:a+4.72 μmol/L PA; g:a+5.5 μmol/L PA**.**

**Fig. S9 UV of CH_3_CH_2_OH -GO-H_2_O_2_-HAuCl_4_ system**

a

g

a:1.13 mol/L CH_3_CH_2_OH+ 3.14 μmol/L CAP+0.193 mmol/L HAuCl_4_ +3.3 mmol/L H_2_O_2_+50ng/mL GO; b:a+0.79 μmol/L PA; c:a+2.36 μmol/L PA; d:a+3.14 μmol/L PA; e:a+4.72 μmol/L PA; f:a+5.5 μmol/L PA; g:a+6.29 μmol/L PA**.**

g

a

**Fig. S10 UV of CH_3_CH_2_OH-GONR-H_2_O_2_-HAuCl_4_ system**

a:1.13 mol/L CH_3_CH_2_OH +0.193 mmol/L HAuCl_4_ +3.3 mmol/L H_2_O_2_; b:a+ 1.58 ng/mL GONR; c:a+6.34 ng/mL GONR; d:a+9.52 ng/mL GONR ; e:a+12.6 ng/mL GONR; f:a+15.8 ng/mL GONR; g:a+23.8 ng/mL GONR

f

a

**Fig.S11 UV spectra of CH_3_CH_2_OH-GO-H_2_O_2_-HAuCl_4_ system**

a:1.13 mol/L CH_3_CH_2_OH+0.193 mmol/L HAuCl_4_ +3.3 mmol/L H_2_O_2_ ; b:a+ 6.6 ng/mL GO; c:a+13.3 ng/mL GO; d:a+20 ng/mL GO ; e:a+26.6 ng/mL GO; f:a+33.3 ng/mL GO.

**Fig.S12a The effect of** GONR **concentration**

1.13 mol/L CH_3_CH_2_OH+17.2 nmol/L Na^+^ + 3.14 μmol/L PA + 0.193 mmol/L HAuCl_4_ + 3.3 mmol/L H_2_O_2_

**Fig. S12b The effect of** PA **concentration**

1.13 mol/L CH_3_CH_2_OH+17.2 nmol/L Na**^+^**+ 0.193 mmol/L HAuCl_4_ +3.3 mmol/L H_2_O_2_+23.8 ng/mL GONR

**Fig. S12c The effect of** H_2_O_2_ **concentration**

1.13 mol/L CH_3_CH_2_OH+17.2 nmol/L Na**^+^**+ 3.14 μmol/L PA+0.193 mmol/L HAuCl_4_ + 23.8 ng/mL GONR

**Fig. S12d The effect of HAuCl_4_ concentration**

1.13 mol/L CH_3_CH_2_OH+17.2 nmol/L Na**^+^**+ 3.14 μmol/L PA+ 3.3 mmol/L H_2_O_2_+23.8 ng/mL GONR

**Fig. S12e The effect of** **temperature**

1.13 mol/L CH_3_CH_2_OH+17.2 nmol/L Na**^+^**+ 3.14 μmol/L PA+0.193 mmol/L HAuCl_4_ +3.3 mmol/L H_2_O_2_+23.8 ng/mL GONR

**Fig. S12f The effect of** **reaction** **time**

1.13 mol/L CH_3_CH_2_OH+17.2 nmol/L Na**^+^**+ 3.14 μmol/L PA+0.193 mmol/L HAuCl_4_ +3.3 mmol/L H_2_O_2_+23.8 ng/mL GONR
